# Induction of acquired drug resistance in endothelial cells and its involvement in anticancer therapy

**DOI:** 10.1186/1756-8722-6-49

**Published:** 2013-07-09

**Authors:** Limin Huang, Christelle Perrault, Jennifer Coelho-Martins, Chaoquan Hu, Charlène Dulong, Mariana Varna, Jielin Liu, Jian Jin, Claudine Soria, Lionel Cazin, Anne Janin, Hong Li, Rémi Varin, He Lu

**Affiliations:** 1 DIFEMA, Merci (EA 3829), Faculté de Médecine et de Pharmacie, Université de Rouen, F-76183 Rouen, France; 2 INSERM, U728-Paris F-75010, France; 3 Sorbonne Paris Cité, Laboratoire de pathologie, UMR-S 728, Université Paris Diderot, Paris F-75010, France; 4 Center of Tissue Engineering and Stem cells, Guiyang Medical University, 550004 Guiyang, China; 5 School of Medicine and Pharmaceutics, Jiangnan University, Wuxi, Jiangsu 214122 China; 6 AP-HP-Hôpital Saint-Louis, Laboratoire de pathologie–Paris, Paris F-75010, France

**Keywords:** Drug resistance, Endothelial cells, ATP-dependent transporter, Anti-cancer therapy

## Abstract

**Background:**

Multidrug resistance (MDR) is one of the major problems in the treatment of cancer. Overcoming it is therefore expected to improve clinical outcomes for cancer patients. MDR is usually characterized by overexpression of ABC (ATP-binding cassette) protein transporters such as P-gp, MRP1, and ABCG2. Though the importance of ABC transporters for cancer cells is recognized, few studies have looked at its implications for the endothelial cells that are essential to tumor angiogenesis. This study investigated the expression and functions of these ABC transporters in endothelial cells *in vitro* and their potential contribution to cancer growth in mice.

**Methods:**

Human micro vessel endothelial cells (HMEC-1) and human umbilical vein endothelial cells (HUVEC) were exposed to increasing doses of Doxorubicin (Dox) to induce ABC gene expression. Cell viability was then quantified by ^3^H-thymidine and MTS assay. Flow cytometry, qPCR, and western blot were used to detect mRNA and the protein expression of P-gp, MRP1, and ABCG2. The intracellular accumulation of Rhodamine 123 (Rho) was used to evaluate drug efflux function and the inhibitors for P-gp, ABCG2, and MRP1 were used to verify their respective roles *in vitro*. In an attempt to evaluate drug resistance in endothelial cells *in vivo*, athymic mice were treated with Dox for 15 days before a MDA-MB-435 tumor graft to observe subsequent changes in the inhibition curves of tumor growth in response to Dox treatment. Furthermore, endothelial cells from multiple sites in these mice were also isolated to estimate their P-gp expression by flow cytometry.

**Results:**

Drug resistance in HMEC-1 and HUVEC was successfully induced by the addition of Dox to the culture media. Two stabilized subcell lines of HMEC1 (HMECd1 and HMECd2) showed 15- and 24-fold increases in resistance. Tests also showed that these induced endothelial cells were cross-resistant to the structurally unrelated drugs Daunorubicin, Vinblastine, and Etoposide. P-gp protein levels increased four and six fold in HMECd1 and HMECd2 as revealed by western blot. The qPCR demonstrated 3.4- and 7.2-fold increases in P-gp, and a slight increase in ABCG2, gene expression. The Rho accumulation within these cells was inversely correlated with the expression levels of P-gp. The inhibitors of P-gp, but not of ABCG2 or MRP1, were able to block the induced endothelial cell resistance to Dox. Furthermore, we also showed that injecting Dox into healthy mice induced an increase in P-gp expression in endothelial cells. Using these pretreated mice in a tumor growth experiment, we observed a dramatic diminution in the therapeutic efficiency of Dox treatment, suggesting implications for drug resistance in mice endothelial cells supporting tumor growth.

**Conclusions:**

ABC transporter expression can be induced in endothelial cells *in vitro*. This study also indicates that P-gp plays an important role in the acquisition of resistance to Dox in endothelial cells and that this reduces the efficiency of chemotherapy.

## Introduction

Recent antitumor drug research has seen the development of a large variety of antiangiogenesis therapies. Because cancer cells in tumors require new blood vessels to grow and spread, they stimulate capillary sprouting from existing vessels and new vessel formation from endothelial precursor cells [1-4]. Recent clinical data shows benefit from the combined administration of antiangiogenic and cytotoxic (chemo- and radiation) therapies, because such combinations target two separate compartments of tumor cancer and endothelial cells. However, recent studies show that antiangiogenic agents also have a direct effect on tumor cells [5,6]. It is also the case that the cytotoxic agents used in chemo- and radiotherapy also affect endothelial cells and inhibit angiogenesis vice versa [7-9].

Drug resistance is an obstacle that impairs the success of cancer therapies. In some cases relapse occurs in initially responsive patients after repeated cycles of chemotherapy due to the acquisition of tumor resistance [10]. Multiple mechanisms contribute to drug resistance, such as increased drug efflux, altered drug metabolism, secondary mutations in drug targets, and the activation of downstream or parallel signal transduction pathways [11,12]. The critical mechanism of cell drug resistance involves the ABC (ATP-binding cassette) protein transporters which pump drug molecules out of cells, leading to reduced effective concentration within them [13]. Well-known ABC transporters include the multidrug resistance (MDR) protein or P-glycoprotein (MDR1, P-gp, ABCB1); the multidrug resistance-associated proteins (MRP1, ABCC1); and the breast cancer resistance proteins (BCRP, ABCG2) [14,15].

P-gp is the first protein to have been shown to be involved in the MDR phenomenon and to be overexpressed primarily in cancer cells [16,17]. It is a protein of 170 kDa containing 1280 amino acids (aa) organized into 12 putative transmembrane domains shared out among two adenosine triphosphate (ATP)-binding cassettes [18,19]. Its role is well established in hepatic drug excretion and limitation of the gastrointestinal absorption of substrate drugs, and as a key component of the blood–brain, blood-testicular, and blood-placental barriers [13,20-24]. It is also expressed in circulating cells such as CD34^+^ hematopoietic progenitor, CD8^+^T cells or natural killer cells [25]. Upregulation of P-gp has previously been shown to increase cancer cells’ ability to efflux a wide variety of structurally unrelated chemotherapeutics such as Vinca alkaloids (Vincristine, Vinblastine), Anthracyclins (Doxorubicin [Dox], Daunorubicin), and Epipodophyllotoxins (Etoposide) [26-28]. Like P-gp, MRP1 and ABCG2 also have wide broad-substrate specificity [29]. All three molecules are reported as being expressed in endothelial cells [30-35].

Several published observations report high level expression of P-gp in tumor endothelial cells [36,37]. In this study, we characterize the induction of a major ABC protein in Human micro vessel endothelial cells (HMEC-1) and human umbilical vein endothelial cells (HUVEC) in response to long-term Doxorubicin treatment. The functional tests are then used to evaluate the protein function. Finally, the athymic mice are treated with Dox to observe the possible occurrence of induced drug resistance in mouse vessels. Our results suggest that P-gp overexpression in endothelial cells could be an early event in the development of chemoresistance and may contribute to the resistant phenotype of tumors *in vivo*. This observation may be helpful when designing novel therapeutic strategies to improve cancer outcomes.

## Materials and methods

### Material

Mouse monoclonal antibodies against human P-gp: C219 were obtained from Calbiochem, La Jolla, CA; 4E3 from Dako, Glostrup, Denmark; and 265/F4 from Abcam, Paris, France. Antibody MRK16 blocking P-gp function was obtained from Kamiya Biomedical Company (Seattle, WA). The anti-ABCG2 antibody BXP-21 came from Abcam and the anti-MRP1 antibody QCRL-1 from Santa Cruz Biotechnology Inc., CA. The antibodies against vWF, flt-1, CD31, or CD105 as well as the FITC or HRP-conjugated F (ab’)_**2**_ fragment of goat anti-mouse IgG were all provided by Dako. Doxorubicin chlorhydrate was purchased from Amersham Pharmacia Biotech (Uppsala, Sweden). Rhodamine 123 and Verapamil were obtained from Calbiochem and Daunorubicin, Etoposide, Vinblastine, Cyclosporine A, Fumitremorgin C, and Diethylstibesterol Terfenadine were provided by Sigma Chemical Co. (Saint Louis, MO).

### Cell culture

Parental and resistant HMEC-1 (Dr TL Lawley, Department of Dermatology, Atlanta) lines were cultured in MCDB-131 medium supplemented with 10% fetal calf serum (FCS), 2 mM L-glutamine, 10 ng/ml EGF, 1 μg/ml hydrocortisone, 100 units/ml penicillin, and 100 μg/ml streptomycin as described elsewhere [38]. Dox-resistant HMEC cells were obtained by continuously exposing cells to escalating concentrations of Dox from 0.001 μg/ml to 0.24 μg/ml over a 12-week period. Two subcell lines of HMEC-1 cells were collected: one was maintained in a culture with 0.08 μg/ml Dox (HMECd1 cells), and another with 0.24 μg/ml Dox (HMECd2 cells). No mutagenic agents were used in the establishment of these Dox-resistant HMEC cells. In the experiments looking at the reversibility of Dox resistance, both HMECd1 and HMECd2 cell lines were cultured in complete medium without Dox for four weeks. HUVEC were isolated as reported elsewhere [39] and seeded on a 1% gelatin-coated plastic flask in MEM-199 medium supplemented with 20% FCS, 15 mM sodium bicarbonate, 15 mM hepes, 2 mM L-glutamine, 10 ng/ml EGF, 1 μg/ml hydrocortisone, 100 units/ml penicillin, and 100 μg/ml streptomycin. Human breast adenocarcinoma cells MDA-MB-435 were cultured in DMEM medium containing 10% FCS, 2 mM sodium pyruvate, 1 mM L-glutamine, 100 units/ml penicillin, and 100 μg/ml streptomycin. All types of cells were digested with trypsin-EDTA once or twice a week and cultured in a 37°C incubator with a 100% humidified atmosphere of 5% CO_2_.

### ^3^H-thymidine Cell proliferation assay

Parental and resistant HMEC sublines were seeded at a density of 4 x 10^4^ cells per well in 48-well culture plates and exposed to a range of drug concentrations for 72 hours at 37°C in an atmosphere of 5% CO_2_. After 70 hours incubation, 1 μCi ^3^H-thymidine (Amersham Pharmacia biotech) was added per well for 2 hours. Wells were then washed twice in PBS and successively incubated with 5% trichloroacetic acid for 20 minutes at 4°C and then 0.5 N NaOH for 90 minutes at 37°C. Radioactivity incorporated into adherent cells was recorded on a **β** counter (Beckman). The 50% cytotoxic concentration (IC50) values were defined as the drug concentration producing 50% inhibition of cell growth and the resistance index (RI) corresponded to the ratio of IC50 values between the resistant and parental cell lines.

### MTS cell proliferation assay

Cell viability was determined using the MTS cell proliferation assay (Promega). Cells grew to a confluence of 90% in 75 cm^2^ cell culture flasks and were passed into 96-well plates (7500 cells/well). Each well contained 100 μl of culture medium supplemented with various concentrations of drugs or with a concentration of DMSO as control. After incubation for either 24, 48, or 72 hours, 20 μl of the MTS reagent was added to each well, and the plate placed in the 5% CO_2_ incubator at 37°C for an additional 2 hours. The optical density (OD) was then read at 492 nm using a microplate reader (Labsystems Multiskan MS). The IC50 values were defined as the concentration of drug producing 50% inhibition of cell growth and the RI corresponded to the ratio of IC50 values between the resistant and parental cell lines. Experiments were performed in triplicate and repeated at least three times.

### Blocking effect assay

P-gp inhibitors Cyclosporine A at 2.5 μM or Verapamil at 1 μM and ABCG2 inhibitors Fumitremorgin C at 5 μM or Diethylstibesterol at 0.5 μM were used in these experiments. After incubation for 48 or 72 hours, cell viability was assessed by the MTS assay. The reversal fold (RF) values, as a measure of the potency of reversal, were obtained from fitting the data to RF = IC50 of cytotoxic drug alone/IC50 of cytotoxic drug in the presence of a modulator [40].

### Rhodamine-123 (Rho) accumulation and efflux assay

HMEC-1, HMECd1, and HMECd2 cells (10^6^/ml in PBS-BSA) were incubated with 1–2 μg/ml Rho in the dark at 37°C in 5% CO_2_ for one hour. Then, the cells were washed twice with ice-cold PBS and analyzed immediately using flow cytometry at different time points. To test Rho efflux specificity, cells were incubated with 30 μM Verapamil or 10 μg/ml MRK16. Results were expressed in an arbitrary unit of the mean fluorescence intensity (MFI). The drug efflux was expressed relative to the amount of drug accumulated.

### Evaluation of mRNA expression via qPCR

HMEC-1, HMECd1, and HMECd2 cells were treated with 2.5 μM Cyclosporine A, 1 μM Verapamil, 5 μM Fumitremorgin C, or 0.5 μM Diethylstibesterol for 24 hours. After incubation, the treated and non-treated cells were harvested and total RNA prepared using the SV total RNA isolation system kit (Promega, USA). The purity of total RNA was checked by a ratio of A260/A280 (>1.9). Total RNA (50 ng) was used to synthesize the first-strand cDNA in a 20 μl reaction solution using the GoScript Reverse Transcription System kit (Promega, USA). Then, 2 μl of cDNA was used for qPCR in triplicates using a taqman® gene expression assay, the primers for P-gp (Hs01067802_m1), ABCG2 (Hs01053790_m1), and the primers for TBP as controls (TATA box binding protein, Hs99999910_m1, Applied Biosystem). The qPCR was performed by 10 minutes of initial denaturation followed by 44 cycles of 15 s at 95°C and 60 s at 60°C in a BioRad CFX96® Real-time System. Delta Ct method was used for analyzing the qPCR results and TBP was used as an internal control for mRNA-level normalization.

### Evaluation of protein expression using western blot analysis

Western blot was performed on whole cell lysates by incubating the cells in the lysis buffer (10 mM Tris pH 6.8, 1 mM EDTA, 10% NP40, 1 mM PMSF, 0.1% SDS) on ice for 30 minutes. Cell debris was removed by centrifugation at 16000 g for 10 minutes. Protein concentration was determined by BCA™ protein assay (Thermo Scientific, USA). A 50 μg protein of each sample was loaded on 8% SDS-PAGE, and the protein transferred to a PVDF membrane by the iBlot™ dry blotting system (Invitrogen, USA). The membranes were blocked by 5% nonfat dry milk for one hour and incubated with either anti-P-gp (Abcam ab-3364) or anti-ABCG2 antibodies (Abcam ab-3380) at 4°C overnight. They were then washed with TBS-tween buffer for one hour and incubated with appropriate HRP-conjugated secondary antibodies (Invitrogen Corp) diluted in blocking buffer for one hour at room temperature. After washing, western blotting luminol reagent (Santa Cruz Biotechnology, USA) was added to the membranes and the chemiluminescence recorded using a Fuji LAS-3000 system. The membranes were then treated with antibody stripping buffer (Gene Bio-application Ltd. Israel), and incubated with anti-actin antibody (1:4000 dilution, Sigma, USA) as control.

### *In vivo* assays

Mice were maintained under specific pathogen-free conditions in the animal facility of the Institut Universitaire d’Hématologie, Saint Louis Hospital in Paris. All experimental procedures were performed in accordance with the recommendations of the European Community (86/609/EEC) and the French National Committee (87/848) for the care and use of laboratory animals. Female athymic nude mice Nu/Nu Swiss (9 weeks of age) (Iffa-credo, France), weighing 18–22 g, were housed under controlled environmental conditions (approximately 25°C) with commercial food and water freely available. Primary results showed that the maximal tolerated dose of Dox by athymic mice for a 6 week period was 6 mg/kg/week. Dox was prepared in 0.9% sodium chloride and ip injections given twice weekly. The experimental procedure consisted of a pretreatment of the mice for 15 days with sodium chloride as a control or 6 mg/kg/week Dox. MDA-MB-435 cells (4×10^6^ cells/200 μl PBS) were then injected subcutaneously into their dorsal midline. Tumor growth was determined 25 days after cell injection and sizes monitored by measuring two diameters with a dial-caliper. Tumor volume was calculated as TV = length × (width)^2^ × π/6.

At the end of the experiments, the mice were sacrificed and the percentage of endothelial cells expressing P-gp on the liver, kidneys, heart, and tumor measured by flow cytometry. Tissues were cut into approximately 1×1-mm^2^ squares and rinsed in physiologic serum. The pieces were incubated with 2 mg/ml collagenase at 37°C for 20 minutes with frequent agitation. The cell suspension obtained following extensive trituration with a 5 ml pipette was filtered on a 70 μm nylon cell strainer followed by a second 40 μm filtration. The second filtrates were centrifuged at 1200 rpm for 5 minutes and the pellets washed twice in 1 ml PBS containing 0.5% BSA. Endothelial cells were isolated by immunoabsorption on magnetic beads coated with anti-mouse CD31 and CD105 IgG according to the recommended protocol (Myltenyi Biotec, France). The isolated cells were characterized by flow cytometry using anti-mouse vWF IgG or C219 antibody. Labeling was revealed by second incubation with fluorescein-conjugated goat anti-mouse IgG.

### Immunohistochemical staining

Immunohistochemical studies were carried out on 5 μm paraffin sections before and after treatment. Primary antibody against P-gp C219 antibody was used at 1:50 dilution. All the immunostainings were performed in an automated immunostainer (Ventana Medical System, France). The intensity and percentage of the cytoplasmic staining on tumor sections were noted.

### Statistical analyses

Data were analyzed using one-way ANOVA and Mann–Whitney U tests as appropriate. The data of qPCR, invasion assay, and *in vivo* data are presented as mean ± SEM. The rest of the data is presented as mean ± SD. A probability value of ≤ 0.05 was regarded as statistically significant.

## Results

### Multidrug resistance of endothelial cells

Our experiments showed that HMEC-1 cells are initially sensitive to Dox treatment. In our attempt to study the induction of drug resistance in endothelial cells, we added progressively increasing doses of Dox into the culture media of the HMEC-1 cells during a period of approximately 12 weeks. When the cells had gradually adapted to the presence of higher concentrations of Dox, two conditions were then chosen to stabilize the Dox-resistant endothelial cell: one population was maintained in a culture with 0.08 μg/ml Dox (HMECd1), and another with 0.24 μg/ml Dox (HMECd2). As shown in Table 1, MTS assay indicated a 15- and 24-fold increase in drug-resistance in the stabilized subcell lines HMECd1 and HMECd2, as compared to their parental cells. ^3^H-thymidine incorporation assay indicated a 36- and 178-fold increase in the RI of HMECd1 and HMECd2 cells in comparison to the parental HMEC cell line (Table 2). Their cellular characteristics were close to those of the parental cells as shown by comparable morphologies and equivalent expression levels of von Willebrand factor, CD31, CD105, flt1, and E-cadherin (data not shown). When we assessed the stability of the Dox-resistant phenotype by culturing HMECd2 in the absence of drugs, we found that after 2 weeks in a drug-free medium, there was no significant change in the drug resistance phenotype or resistance index. However, when grown without selection pressure for 4 weeks, the RI to Dox decreased from 178.5 to 1.25 (p < 0.001). Therefore, endothelial cells were able to induce or reverse the expression of P-gp.

**Table 1:** Modulation of drug resistance to Dox by Verapamil and Cyclosporine A in HMECd1 and HMECd2

	**HMEC-1**	**HMECd1**			**HMECd2**		
**Agents**	**IC50 (μM)**	**IC50 (μM)**	**RI**	**RF**	**IC50 (μM)**	**RI**	**RF**
**Dox**	**0.052 ± 0.001**	**0.785 ± 0.049**	15.09*	**1.00**	**1.257 ± 0.055**	24.17*	**1.00**
**+ Vrp 1 μM**	**0.051 ± 0.002**	**0.386 ± 0.075**	7.56*	1.99*	**0.225 ± 0.062**	4.41*	5.59*
**+ CysA 2.5μM**	**0.049 ± 0.004**	**0.251 ± 0.041**	5.12*	3.13*	**0.159 ± 0.057**	3.24*	7.91*

The cells were treated as described and tested by MTS assay. The resistance index (RI) was determined as the IC50 of Dox-treated HMECd1 or HMECd2 cells divided by the IC50 of Dox-treated HMEC-1 cells. The resistance fold (RF) was calculated as the IC50 of Dox-treated HMECd1 or HMECd2 cells over the IC50 of the same cell line as treated by Dox plus P-gp inhibitors. *p <0.05 for statistical significance.

**Table 2:** Cross-resistance of HMECd1 and HMECd2

	**IC50 μg/ml (RI)**		
	**HMEC-1**	**HMECd1**	**HMECd2**
**Doxorubicin**	**0.0028 ± 0.0003 *(1)***	0.1 ± 0.027 (35.7)*	0.5 ± 0.01 (178.5)*
**Daunorubicin**	**0.018 ± 0.009 *(1)***	0.92 ± 0.01 (51.1)*	1.7 ± 0.78 (94.4)*
**Vinblastine**	**0.023 ± 0.007 *(1)***	0.16 ± 0.03 (69.5)*	0.2 ± 0.03 (86.9)*
**Etoposide**	**0.0031 ± 0.0006 *(1)***	0.062 ± 0.0062 (20)*	0.6 ± 0.094 (203.5)*
**Mytomycin C**	**0.16 ± 0.0074 *(1)***	**0.15 ± 0.071 *(0.9)***	**0.18 ± 0.0003 *(1.1)***

The cells were cultured for 72 hours at 37°C in the presence of increasing concentrations of Dox, Etoposide, Daunorubicin, Mytomycin C, or Vinblastine, then incubated with 1 μCi ^3^H-thymidine/well for 1 hour at 37°C. The radioactivity incorporated into the cell was then measured. IC50 were determined over three separate experiments. Results are expressed as the mean ± SEM. *p <0.05 for statistical significance.

The resistance of these cells to other drugs was then tested. The use of three MDR-related drugs, Daunorubicin, Vinblastine, or Etoposide, showed that both of the Dox-resistant endothelial cell lines were also resistant to higher concentrations of these drugs compared to parental cells (Table 2). In contrast, no significant differences between parental and resistant sublines were found with Mytomycin C treatment (Table 2).

### P-gp is predominantly expressed in the resistance of endothelial cells

**Figure 1:**
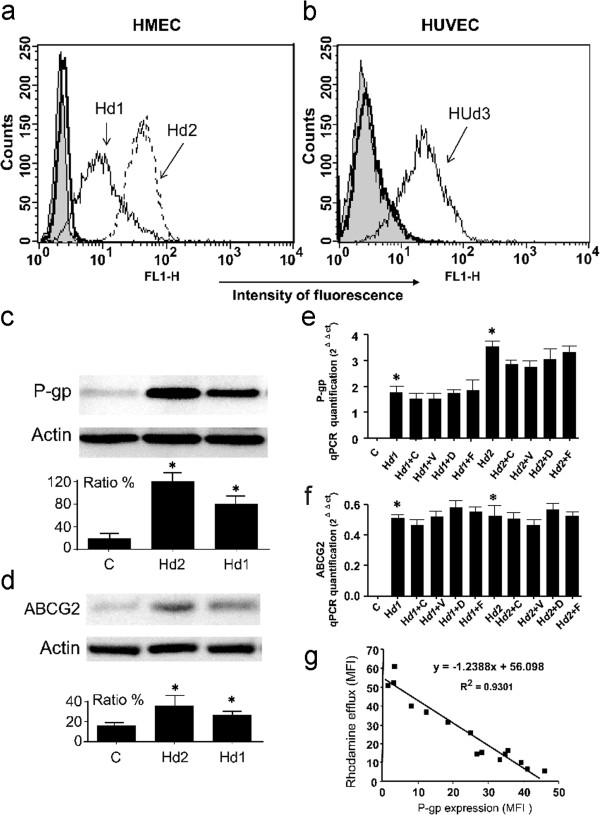
**Induced P-gp-mediated drug resistance of endothelial cells.** P-gp cell surface expression was analyzed with flow cytometry in HMEC (Panel **a**) or HUVEC cells (Panel **b**). Endothelial cells expressed P-gp after the induction by Dox treatment. Parental (thick black line), HMECd1 and HUVECd3 (thin black line), and HMECd2 (−−) cells were incubated with 10 μg/ml 4E3. Incubation with control IgG2a gave similar histograms for the three cell lines (filled grey histogram). Histograms are representative of four separate experiments. Panel **c**: The western blot of P-gp levels in HMECd1, HMECd2 and their parental cells. The data for the ratio were obtained with three repeated blots. *: p < 0.05 in comparison with the controls. Panel **d**: The western blot of ABCG2 levels in these cells. The data for the ratio were obtained with three repeated blots. *: p < 0.05 versus the controls. Panel **e**: qPCR (primer Hs01067802_m1) results of P-gp mRNA levels in treated or nontreated HMEC-1, HMECd1, and HMECd2. Cyclosporine A (C), Verapamil (V), Fumitremorgin C (F), and Diethylstibesterol (D) were used to treat the cells. The results were obtained from three independent experiments. *: p < 0.05 versus the nontreated cells. Panel **f**: qPCR (Hs01053790_m1) results of ABCG2 mRNA levels in treated or nontreated HMEC-1, HMECd1, and HMECd2. Cyclosporine A (C), Verapamil (V), Fumitremorgin C (F), and Diethylstibesterol (D) were used to treat the cells. The results were obtained from three independent experiments. *: p < 0.05 versus the nontreated cells. Panel **g**: Correlation between P-gp surface expression and its efflux function. During the establishment of resistant HMEC cell lines, the P-gp surface expression and the Rho efflux were regularly analyzed by flow cytometry, as shown in Figure 2a-d (R^2^ = 0.9301).

Flow cytometric studies demonstrated a high level of P-gp expression on the cell surface of Dox-treated cells, whereas it was almost absent on parental cells (Figure 1a and b). P-gp surface expression was dependent on the Dox concentration used for cell establishment; it reached 9.2 ± 2.9 MFI for HMECd1 cells (p < 0.05) and 45.1 ± 8.4 MFI for HMECd2 cells (p < 0.005) compared with 2.8 ± 0.8 MFI for parental cells. This P-gp expression represented a 3.2- and 16-fold increase in comparison with parental cells. Interestingly, when treated with 0.16 μg/ml Dox for 15 days, a primary culture of endothelial cells isolated from the human umbilical vein also expressed a P-gp protein on their surface (Figure 1b). In contrast, we did not find any expression of MRP1 in both Dox-resistant HMEC and HUVEC (data not shown)

Western blot analysis of the levels of P-gp showed that its expression in drug-resistant HMECd1 and HMECd2 cells increased about 4- and 6- fold, respectively (Figure 1c). Furthermore, we also determined the changes of P-gp mRNA levels using qPCR. The results showed an increase in P-gp mRNA by approximately 3.4 and 7.2 folds in HMECd1 and HMECd2 cells, respectively regardless of the presence of the P-gp or ABCG2 inhibitors (Figure 1e).

Levels of ABCG2 expression on drug-resistant HMECd1 and HMECd2 cells were also evaluated using qPCR and western blot. Our results showed a 1.41- and 1.68-fold increase in ABCG2 mRNA in HMECd1 and HMECd2 cells, regardless of the presence of the ABCG2 or P-gp inhibitors (Figure 1f). The ABCG2 protein also increased about 1.5 and 2 fold, respectively (Figure 1d). Thus, our results indicate that Dox induced predominantly P-gp expression.

### Dox-induced P-gp mediates endothelial cells’ resistance to Dox

**Figure 2:**
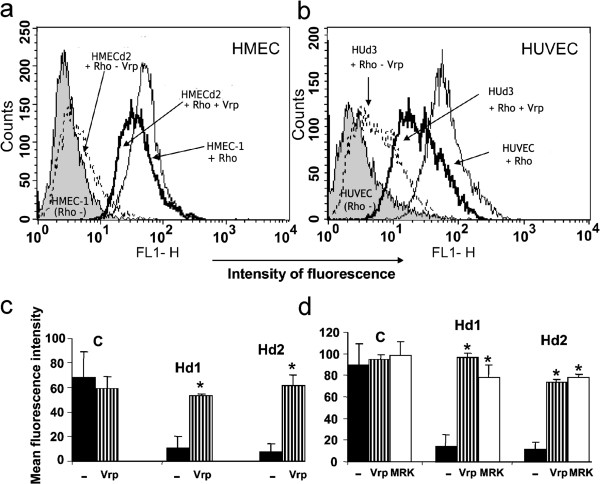
**Endothelial cells express functional P-gp protein.** Panel **a** and **b**: Verapamil blocks Rho efflux in endothelial cells. Dox- or noninduced HMEC and HUVEC cells were incubated in the absence or presence of Rho for 1 hour at 37°C. Rho accumulation was inhibited by the addition of Verapamil (Vrp) at 30 μM to the cell incubation mixture. The cells were analyzed in the flow cytometer. Panel **c** and **d**: Activity of endothelial P-gp is blocked by Verapamil and MRK16. Parental HMEC, HMECd1, and HMECd2 cells were incubated with 1 μM **(c)** or 2 μM **(d)** Rho for 1 hour at 37°C in the absence or presence of 30 μM Verapamil or 10 μg/ml MRK16 (MRK). Rho accumulation was measured by flow cytometry and quantified as the MFI. The background fluorescence level, determined using cells not exposed to Rho, was subtracted from the data. Results are expressed as the mean±SEM of 3 separate experiments. *p < 0.025, **p < 0.01.

Transporter functionality was tested by evaluating the ability of these cells to efflux a fluorescent Rho probe. Kinetic analyses by flow cytometry showed that parental cells incorporated the fluorescent probe in a time-dependent manner, reaching a plateau of 41.2 ± 7.9 MFI at 80 minutes (result not shown). In contrast, both Dox-resistant cell lines demonstrated a significant decrease in Rho accumulation (indicative of an enhanced efflux), reaching 13.1 ± 3.9 MFI for HMECd1 and 6.9 ± 1.3 MFI for HMECd2 at 80 minutes (p < 0.025). This indicated a 68% and 83% reduction in intracellular Rho accumulation (Figure 2a). Similar experiments with Dox-treated and untreated HUVECs showed that only the former could significantly and specifically efflux Rho (p < 0.05) (Figure 2b). When incubating both Dox-resistant HMEC cells in the presence of 5 μM Rho for one hour at +4°C, to block the energy-dependent function of P-gp, the Rho uptake reached ≈ 34.5 MFI, a comparable value to that of 38.4 ± 3.3 MFI obtained for parental cells. By analyzing data obtained during the establishment of Dox resistance, we demonstrated a linear correlation between P-gp transporter expression and its Rho efflux function as confirmed by a correlation factor R^2^ of 0.9301 (Figure 1g), indicating P-gp plays a major role in drug efflux in these cells.

### Blocking P-gp attenuates the resistance of endothelial cells to Dox

We tested the effects of two functional inhibitors of P-gp, Verapamil and the MoAb MRK16, on Rho accumulation (Figure 2a-d). The presence of Verapamil did not significantly modify the Rho accumulation in parental HMEC cells (Figure 2c, d). In contrast, it effectively blocked efflux of the fluorescent dye in both Dox-resistant cell lines, raising them significantly to an intracellular Rho level comparable to that of parental HMECs (Figure 2c, d). The very low Rho accumulation (2 μM) in HMECd1 and HMECd2 cells increased to 96.1 ± 4.9 MFI (p < 0.01) and 73.45 ± 2.5MFI (p < 0.025) respectively when 30 μM Verapamil was added. Varying the concentration of Verapamil from 1 to 100 μM resulted in a progressive increase of intracellular Rho accumulation, indicating its specific effect. This reached a plateau at 30 μM (data not shown). The presence of the specific P-gp inhibitory MoAb, MRK16, reproduced the effect of Verapamil and restored a level of Rho accumulation in both HMECd1 and HMECd2 similar to that of parental cells (Figure 2d). In contrast, QCRL-1, a MoAb directed against MRP1, had no effect on Rho accumulation (data not shown). Taken together, these results indicate that the loss of Rho accumulation in Dox-resistant endothelial cells involves the P-gp function which has the property to mediate cell exclusion of drugs.

We then checked cell survival after Dox treatment in the presence of Cyclosporine A and Verapamil in both HMECd1 and HMECd2 cells. The cells were treated with a series of Dox concentrations in the presence of 2.5 μM Cyclosporine A or 1 μM Verapamil (that blocks the P-gp function). The results clearly show that the blockage of the P-gp function restored the sensitivity of HMECd1 and HMECd2 cells to Dox (Table 1). In contrast, the ABCG2 inhibitors Fumitremorgin C and Diethylstibesterol had no such effect (data not show). Therefore, our results suggest that P-gp plays a major role in the acquisition of Dox resistance in HMECd1 and HMECd2.

### Involvement of endothelial P-gp in tumor drug resistance

**Figure 3:**
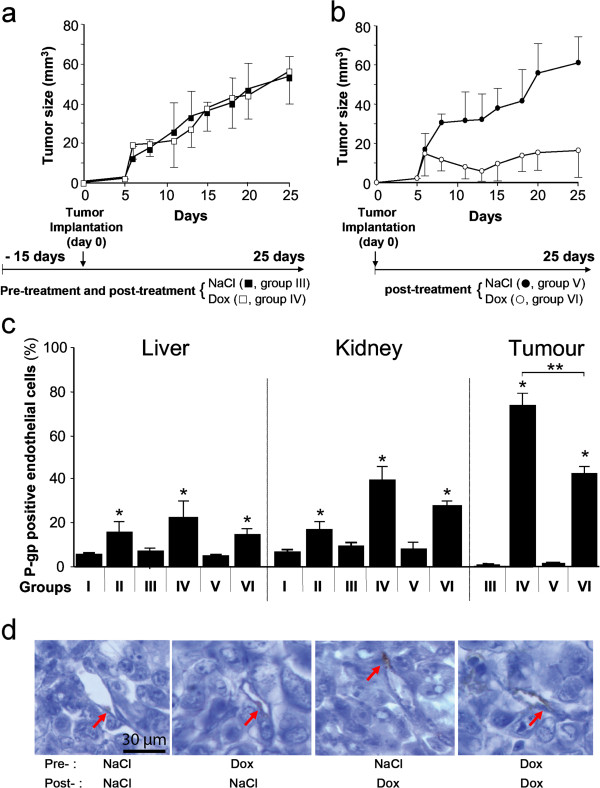
**Induction of P-gp expression and its involvement in tumor growth in mice.** Panel **a**. Dox pretreatment before tumor xenograft inhibited the therapeutic efficiency of Dox therapy. Athymic mice were pretreated for 15 days with either NaCl (group III) or Dox (group IV). Injection of MDA-MB-435 cells was performed subcutaneously in each mouse and the treatment (NaCl for group III and Dox for group IV) was administered for 25 days. Panel **b**. Therapeutic efficiency was observed in Dox-treated mice who had not received Dox pretreatment. The same experiment was performed without the 15-day pretreatment in mice receiving only NaCl (group V) or Dox (group VI). Results are expressed as the mean ± SEM of 10 mice per group. Significant difference in tumor growth rates was found between groups V and VI (*p < 0.05), but not between groups III and IV. Panel **c**: Presence of endothelial P-gp in the organs of Dox-treated mice. Livers, kidneys, and tumors from the six groups of treated athymic mice were removed. Following digestion with collagenase, cell suspensions were filtered and washed in PBS-BSA. Endothelial cells were isolated and characterized by flow cytometry using 10 μg/ml of control IgG or C219 antibody. The histograms represent the percentage of endothelial cells positive for P-gp. Results are expressed as the mean ± SEM with 10 mice in each group and the experiments were repeated at least 3 times. * : P < 0.05 in comparison to the control groups I or III without Dox treatment; ** : p < 0.05 between group IV versus group VI. Panel **d**: Immunochemical staining of P-gp on the tumor sections. Red arrows indicate endothelial cells with lumen within the tumors. The tumors were obtained and sectioned at the end of the experiments as described in above panels.

To evaluate the role of endothelial P-gp in tumor protection, we also tested its influence on tumor growth *in vivo*. Two groups of athymic nude mice were pretreated with intraperitoneal (ip) Dox injection of 6 mg/kg/week over a 15-day period (groups II and IV) whereas groups I and III were injected with physiologic serum. Groups V and VI had no pretreatment. The Dox prescription corresponded to the maximum well-tolerated dose of Dox and resulted in barely 4-8% body weight loss during the experiment and no deaths. MDA-MB-435 cells were then subcutaneously inoculated in the dorsal midline (groups III to VI). The posttreatment began, corresponding to the physiologic serum (groups I, III, V) or Dox (II, IV, VI) injections. The data in Figure 3a and 3b show the tumor growth evolution for the different treatments. When injected after tumor implantation, Dox effectively inhibited tumor growth, reaching 16.4 ± 13.9 mm^3^ at 25 days, a 3.7-fold decrease in tumor size compared to mice receiving physiologic serum (60.8 ± 13.5 mm^3^, p < 0.025) (Figure 3b). In contrast, when the mice had been sensitized by Dox injection for 15 days before tumor implantation, the tumors responded poorly to Dox posttreatment and we found a comparable tumor growth between mice receiving Dox (55.9 ± 16.1 mm^3^) and physiologic serum (53.2 ± 10.3 mm^3^) throughout the experiment (Figure 3a). To better understand the ineffectiveness of Dox pretreatment on the blockage of tumor growth, we sacrificed animals and performed histological and flow cytometric studies after cell dissociation of liver, kidneys, heart, and tumor for ten mice in each group. This showed that the Dox treatment given either post- or pre- and posttreatment did not significantly modify the morphology of these organs. In particular, no sign of cardiotoxicity was observed across the different groups.

To better quantify the P-gp positive endothelial cells in the mice, we removed their organs and tumors. Following cell dissociation, the isolated endothelial cells were characterized by flow cytometric analysis and the percentage of endothelial cells positive for P-gp labeling (C219) was measured (Figure 3c). Our results show that for the liver and kidney, 10-40% of endothelial cells became positive for P-gp expression following Dox pre- and/or posttreatment (groups II, IV, and VI). Endothelial cells within the tumor acquired the resistant phenotype when the animals had been treated with Dox (group VI, 44.74 ± 3.55%) in comparison with buffer administration (groups III and V, ~1%). When Dox was administered as a pretreatment in group IV, the percentage of P-gp positive endothelial cells within the tumor reached the remarkable level of 78.01 ± 6.39%. Immunohistological observation of these tumor sections at the end of the experiments demonstrated an induced P-gp staining on the endothelial cells, and no evident induced P-gp staining in the surrounding tumor cells (Figure 3d). These data suggest that endothelial cells participate in the resistant phenotype of tumors by serving as an initial barrier between chemotherapeutics and tumor cells.

## Discussion

This study was designed to evaluate the expression of P-gp, MRP1, and ABCG2 and their activities in endothelial cells after cell exposure to Dox. We have shown for the first time that P-gp expression was upregulated in two stabilized Dox-resistant endothelial cells, HMECd1 and HMECd2. P-gp protein levels revealed by western blots were found to have increased 4- and 6- fold in both HMECd1 and HMECd2 cells. Similarly, the qPCR experiment demonstrated 3.4 and 7.2 fold increases in P-gp gene expression. The functional efflux test using Rho 123 demonstrates a linear correlation between P-gp transporter expression and efflux function. We further show that the drug spectrum of P-gp-mediated drug resistance corresponded to the P-gp functional character and that the blockage of P-gp activity by the P-gp inhibitors Verapamil and Cyclosporine A attenuated the cells’ capacity for Dox resistance. Furthermore, we demonstrate that the resistant cell phonotype induced by Dox treatment can be slowly reversed after withdrawal of the drug in culture.

We studied ABCG2 because it is another well-known ABC transporter used to efflux a wide variety of substrates, in particular some anticancer drugs such as Mitoxantrone, Doxorubicin, and Daunorubicin [29,41]. We observed a significant induction of ABCG2 expression in HMECd1 and HMECd2, though this was much less pronounced than that of P-gp. Since both inhibitors of ABCG2 (Fumitremorgin C and Diethylstibesterol) failed to reverse Dox resistance in HMECd1 and HMECd2, this also suggests that the drug efflux in HMECd1 and HMECd2 was due to the upregulated P-gp level. MRP1 was also evaluated in this study. However, neither western blot nor flow cytometry detected its significant expression in noninduced cells nor was there an increase in expression in the induced cells. Accordingly, the anti-MRP1 antibody QCRL-1 MoAb had no effect on cell survival. Although ABCG2 and MRP1 were shown not to be functionally responsible for the drug resistance observed here, the possibility that they may play important roles in the drug resistance of endothelial cells in other circumstances cannot be excluded [34,35,42].

Recent studies have emphasized the importance of tumor vasculature and an appropriate pressure gradient for adequate drug delivery to the tumor [43-45]. In addition, some cancer cells that are sensitive to chemotherapy in cultured cell monolayers become resistant when transplanted into animal models. This indicates that environmental factors such as the extracellular matrix or tumor geometry might be involved in tumor drug resistance [46].

Our data also give rise to questions about the involvement of acquired P-gp expression on endothelial cells in tumor resistance. To induce P-gp upregulation, we firstly treated the mice with Dox before tumor implantation. The results of the immunostaining and cytometry analysis of the isolation of endothelial cells shown in Figure 3 demonstrate significantly higher P-gp expression in the livers and kidneys of the treated mice, confirming the rapid response of normal endothelial cells to Dox challenge. These observations are in agreement with the tissue distribution of P-gp [47]. We further isolated the endothelial cells from the tumors, and the results clearly demonstrated a higher expression of P-gp on the tumor vessels after Dox treatment. The highest expression of P-gp was found in those mice that had been treated with Dox before tumor implantation, whereas positive, but less stained, endothelial cells were observed in the short treatment groups, compared to the negative control mice. Immunochemical staining of the tumor sections confirmed the result. These results indicate that normal vessels as well as tumor vessels react to Dox injection. Our results are also consistent with recent studies showing that endothelial cells isolated from human tumors are less sensitive to anticancer drugs [28,48].

To evaluate the effect of the acquired Dox resistance of endothelial cells on tumor growth in preclinical models, we also evaluated tumor growth in the mice where such resistance had been induced. The results demonstrated that Dox has an inhibitory effect on MDA-MB-435 tumor growth transplanted into control nude mice. In the mice that had been pretreated by Dox before tumor graft, tumor growth continued and responded poorly to Dox treatment. Acquired resistance to Dox in the pretreated group is believed to greatly reduce the anti-cancer efficacy of Dox. Importantly, as demonstrated in this model by P-gp immune staining of the tumor sections, upregulation of P-gp expression after Dox treatment was found essentially in tumor endothelial cells, but not in tumor cells themselves. Therefore, these results strongly suggest that acquired resistance in tumor endothelial cells plays a role in the overall therapeutic response to anticancer drugs.

Taken together, these findings underline the importance of drug resistance in endothelial cells in both *in vitro* and *in vivo* experiments. Recent reports provided evidence for acquired drug resistance in tumor endothelial cells in cancer patients [36,37]. We believe that further investigation of this aspect will be helpful in understanding the complex mechanisms of MDR in cancer. We hope that circumventing endothelial cell drug resistance may improve conventional chemo- and antiangiogenic therapies.

## Abbreviations

MDR: Multidrug resistance; P-gp: P-glycoprotein; MRP1: Multidrug resistance-associated proteins; ABCG2: Breast cancer resistance protein; HMEC-1: Human micro vessel endothelial cell; HUVEC: Human umbilical vein endothelial cells; qPCR: Quantitative polymerase chain reaction.

## Competing interests

The authors declare that they have no competing interests.
